# Can a Checklist Improve the Informed Consent Process?

**DOI:** 10.7759/cureus.13148

**Published:** 2021-02-05

**Authors:** Eric Shirley, Veronica H Mai, Kevin M Neal, Kathryn V Blake

**Affiliations:** 1 Orthopaedics, Naval Medical Center Portsmouth, Portsmouth, USA; 2 Orthopaedics and Rehabilitation, Lincoln Memorial University-DeBusk College of Osteopathic Medicine, Harrogate, USA; 3 Orthopaedics, Nemours Children's Health System, Jacksonville, USA; 4 Pediatrics, Nemours Children's Health System, Jacksonville, USA

**Keywords:** informed consent, checklist, shared decision making

## Abstract

Informed consent often fails to provide patients and families with a full understanding of the proposed procedure. We developed an informed consent checklist for identifying specific aspects of the surgical consent that were not fully understood by families. The purpose of this study was to measure the effect of using this checklist on families’ knowledge, satisfaction, experience, and decisional conflict during the consent process. The families of pediatric patients scheduled for an orthopaedic preoperative visit were prospectively randomized into one of two groups: checklist or traditional appointment. Families in the checklist group completed the informed consent checklist which was then used by the surgeon to further discuss aspects of the surgery that needed clarification. Those in the traditional group had similar discussions about surgery without the aid of a checklist. Sixty-one families participated in the study; 27 in the checklist group and 34 in the traditional group without a checklist. The checklist group reported no difference in mean scores for all satisfaction (P = 0.37), decisional conflict (P = 0.51), and knowledge items (P = 0.31). For patient experience, the traditional group reported the visits were significantly more relaxed (mean 4.9, 95% confidence interval (CI) 4.8-5.0) than the checklist group (mean 4.5, 95% CI 4.3-4.7). Our results suggest that having a family member complete the informed consent checklist prior to meeting with the surgeon did not improve, and may worsen, the consent experience for some families. Other methods need to be evaluated to determine the optimal consent process from the family’s perspective.

## Introduction

The informed consent process can be divided into three stages: physician disclosure, patient understanding, and decision making [1]. The disclosure stage rarely meets existing criteria for proper consent due to improper explanations of treatment goals, benefits, or risks in a retainable manner [2-8]. Patient or family understanding is often subsequently assessed using only a single question such as “Do you understand?”, “Are there any questions?”, or even “Are you ready to sign the consent?” Decision making is impaired as adequate understanding of informed consent for surgery is achieved in less than one-third of patients [9].

The checklist is a simple tool used to improve task completion, recall important information, and process standardization. Checklists have been used in hospital settings to increase adherence to clinical pathways, task completion during ward rounds, and patient safety in the operating room [10-12]. We developed a preoperative informed consent checklist to improve this process that identified aspects about the surgery that were unclear to families so that clarification could be provided. The objective of this study was to measure the effect of using this checklist on knowledge, satisfaction, experience, and decisional conflict during the informed consent process at a preoperative visit. Our hypothesis was that checklist use would result in improvements in these domains compared to families who were not offered the checklist.

## Materials and methods

Participants

Pediatric patients (17 years or younger) and their family members (parent/guardian) who presented for a preoperative visit with either of two pediatric orthopaedic surgeons were invited to participate in the study. The purpose of this preoperative visit was to review the information provided at a previous visit when the surgery was proposed, perform a history/physical, and obtain informed consent. These preoperative visits were scheduled within 30 days of surgery in order to minimize delays and cancellations on the day of surgery. Patients with problems requiring more urgent treatment (i.e. fractures) where surgery was planned at the initial encounter were excluded. 

Study design and measurement

**Figure 1:**
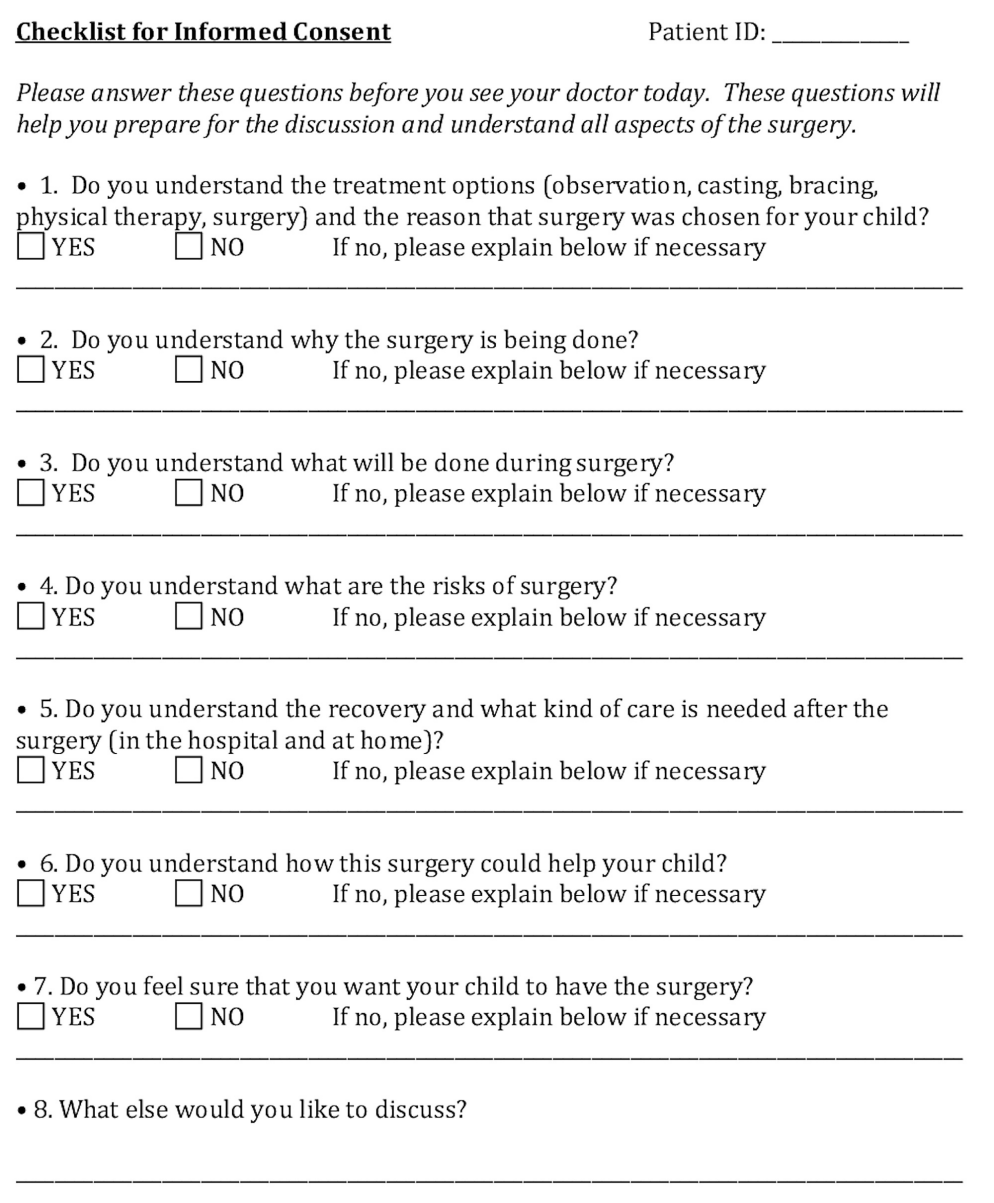
Checklist completed by parents/guardians in the checklist group

An eight-item preoperative visit checklist was developed by one of the pediatric orthopaedists and a member of the institution’s Investigational Review Board experienced in decision-making research (Figure 1). The items confirmed understanding of discrete components of the surgical consent (options, indications, technique, recovery, risks, benefits), and also asked the question “Do you feel sure that you want your child to have the surgery?” to assess whether families were uncertain about their decision to proceed with surgery [13]. Options to each item were “yes” or “no”; space was provided for elaboration of any “no” response. The form was designed to be completed by families and subsequently used as a checklist by the surgeon in order to further discuss these components and to identify families who remained unsure about their child having surgery.

**Figure 2:**
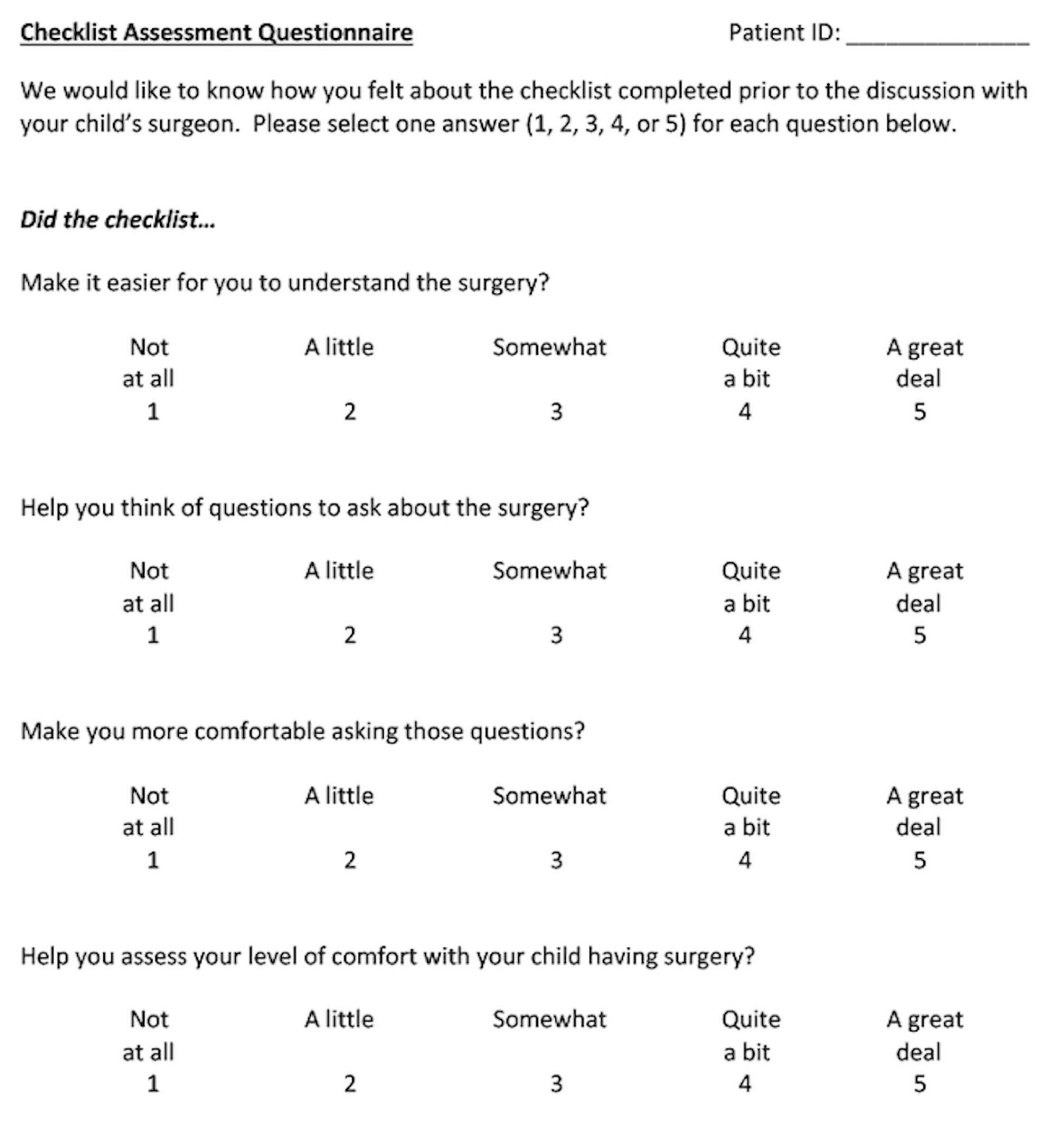
Checklist assessment questionnaire completed by parents/guardians

A four-item checklist assessment questionnaire was utilized to assess the parent/guardian’s impression of the checklist (Figure 2). This questionnaire was created for this study because a validated metric for checklist use had not been developed. The survey contained a 5-point Likert scale, with a score of 5 on each item reflecting a positive view of the checklist (maximum possible points, 20). 

An 11-item visit survey was developed in order to assess families’ knowledge, satisfaction, patient experience, and decisional conflict during the preoperative visit. The decisional conflict domain was included as this represents a patient’s uncertainty when faced with choices involving risk, loss, regret, or a challenge to values [14]. Responses to each item also utilized a 5-point Likert scale ranging from “Not at all” (1 point) to “A great deal” (5 points) (maximum possible points, 55). This survey was created for this study because a comprehensive validated measurement tool for the consent process in pediatric patients had also not been developed. 

Procedure

**Figure 3:**
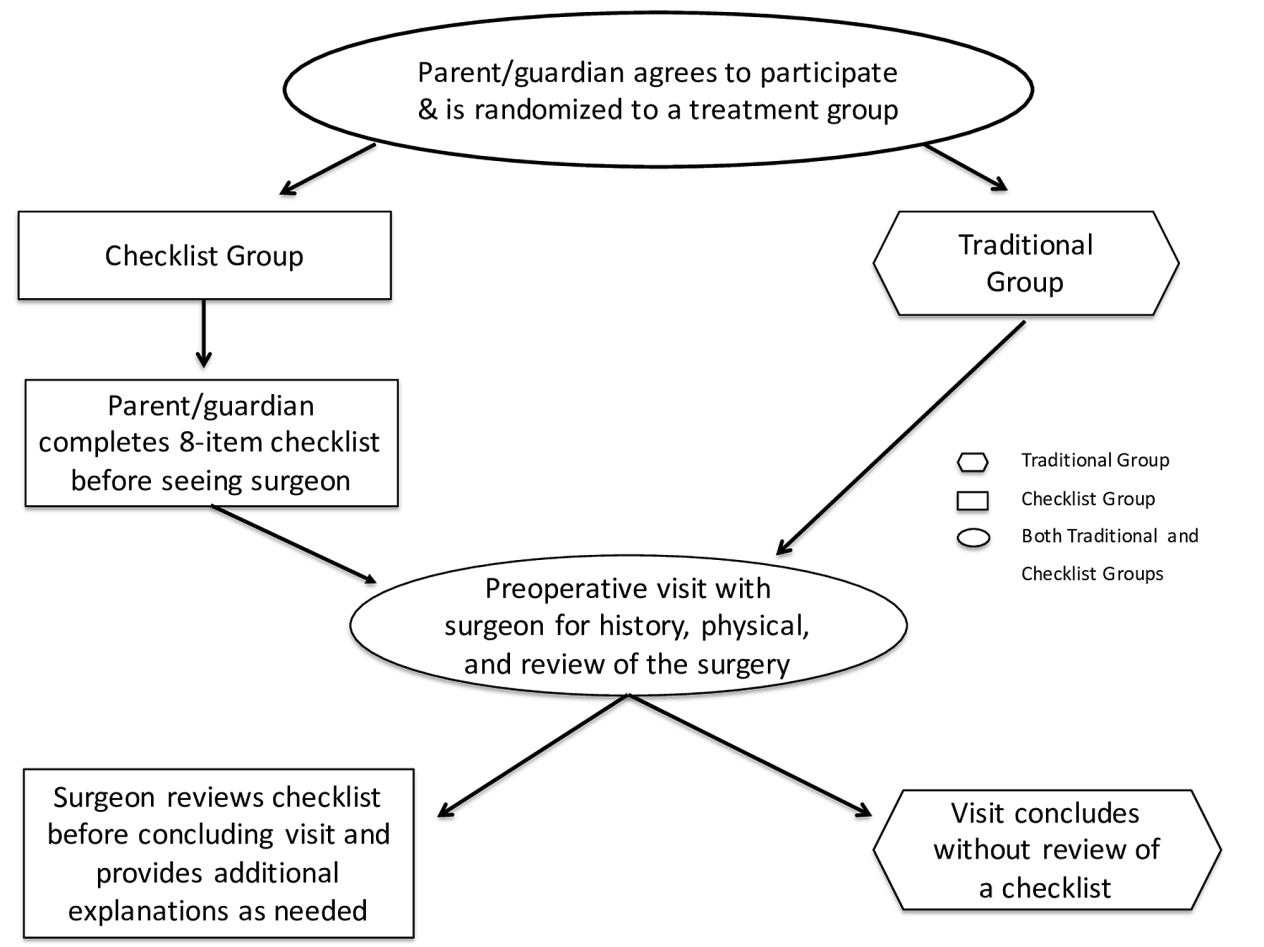
Consent process for traditional and checklist groups

Families were enrolled after obtaining Institutional Review Board approval for the study. A research assistant explained the study to eligible patients at preoperative visits held at a tertiary medical center’s main campus location. No compensation was provided. Families were then prospectively randomized by choosing a sealed blank envelope that placed them into one of two consent process groups: a traditional group (without a preoperative checklist) or a checklist group (with a preoperative checklist) (Figure 3). 

In the checklist group, all families received and completed the checklist in the examination room before seeing the surgeon. An additional checklist was not utilized when more than one parent/guardian was present. The surgeon first performed a history and physical and reviewed the surgery with the family. The study envelope was then opened and the surgeon completed the discussion by reviewing each checklist item with the family and providing clarification to checked items which indicated they did not fully understand. 

In the traditional group, the surgeon also performed a history and physical and reviewed the surgery with the family. The study envelope which did not contain the checklist was then opened and the surgeon subsequently verified understanding of the procedure in their routine manner. 

After the appointment, participants in both groups completed the 11-item visit survey. A single survey was used for each patient regardless of the number of family members present. Families in the checklist group also completed the checklist assessment questionnaire. Both outcomes measures were collected on the day of the preoperative visit. 

Analysis plan

Patients were enrolled over a six-month period. Statistical analysis consisted of Wilcoxon rank-sum tests and unpaired t tests to compare the visit survey results between the groups using GraphPad Prism Software (GraphPad Software Inc., San Diego, CA). Significance was set at P < 0.05. Additional analyses were descriptive based on examination of the distributions and confidence intervals of the survey scores.

## Results

**Figure 4:**
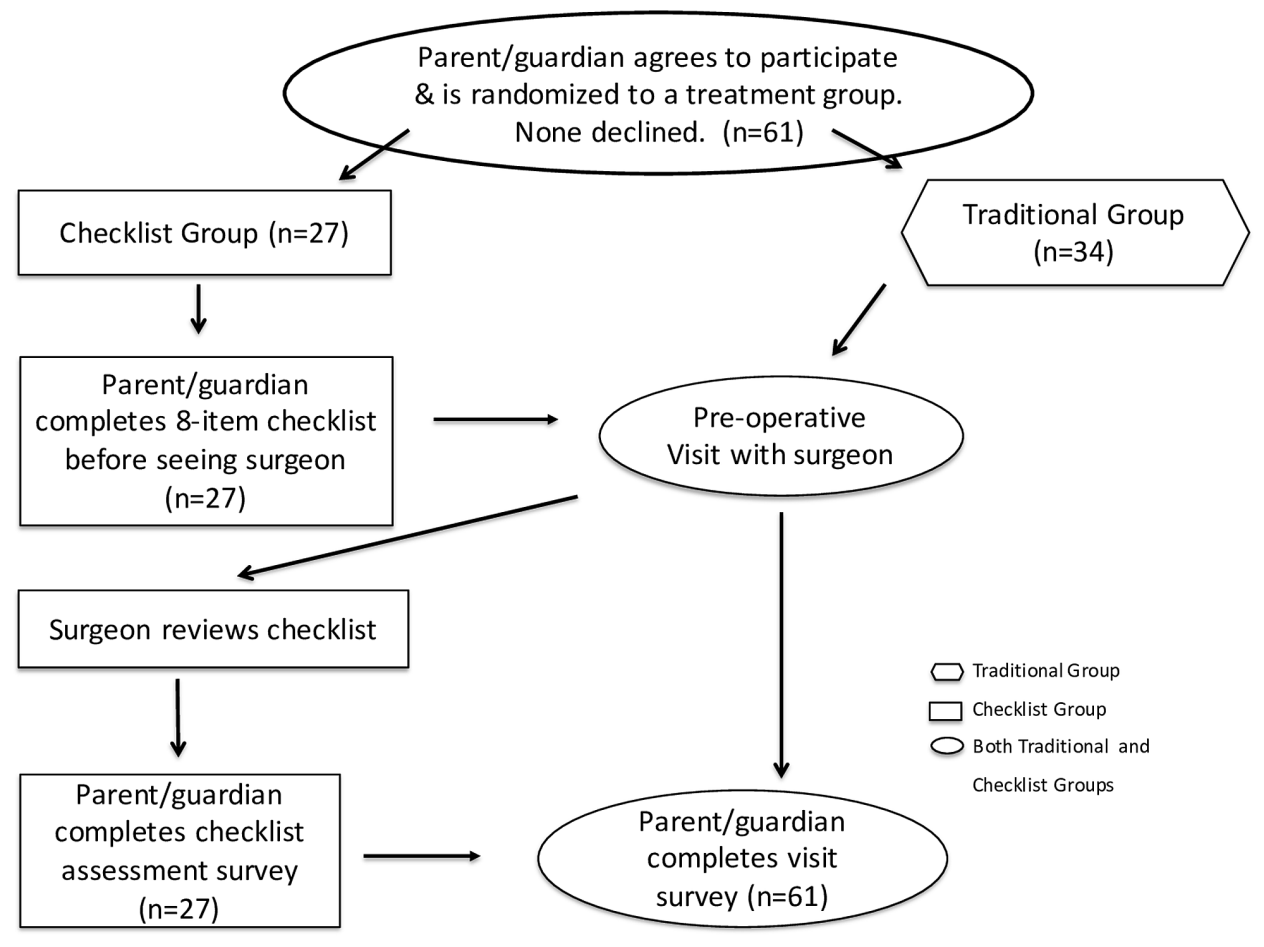
Consort diagram of families offered participation in the study

Families of 61 patients participated in the study; 27 in the pre-appointment checklist group and 34 in the traditional group without a pre-appointment checklist (Figure 4). Both groups had patients who were to undergo anterior cruciate ligament reconstruction, proximal femur osteotomy, muscle lengthening surgery for spasticity, or posterior spinal fusion. All families approached agreed to proceed, completed the study protocol, and were analyzed by intention to treat. 

On the informed consent checklist; 9/27 (33.3%) of families had questions regarding recovery, 7/27 (25.9%) about surgical risks, 7/27 (25.9%) about what is done during surgery, and 3/27 (11.1%) about alternatives to surgery. One family (1/27, 3.7%) indicated they were not sure about wanting their child to have the surgery. All 27 families (100%) completing the pre-appointment checklist were clear about why the surgery was being done and how the surgery would help their child. 

The mean total score of the checklist assessment questionnaire was 17.3 (95% confidence interval (CI), 15.9-18.7); 25/27 (92.6%) of the families provided total scores of 14 or higher, whereas 2/27 (7.4%) provided a total score of 7. The mean scores for the four items were 4.4 (95% CI, 4.1-4.7) for making families more comfortable with asking questions, 4.3 (95% CI, 3.9-4.7) for making it easier to understand the surgery, 4.3 (95% CI, 3.9-4.7) for helping to think of questions to ask, and 4.3 (95% CI, 3.9-4.7) for assessing one’s level of comfort with the child having surgery.

The overall mean visit survey total score was 53.4 (95% CI, 52.3-54.5) in the traditional group and 52.2 (95% CI, 50.8-53.6) in the checklist group (Table 1). The checklist group reported no significant difference in mean scores for all satisfaction (P = 0.37), decisional conflict (P = 0.51), and knowledge items (P = 0.31). For patient experience, the traditional group reported the visits were significantly more relaxed (mean 4.9, 95% CI 4.8-5.0) than the checklist group (mean 4.5, 95% CI 4.3-4.7). There were no significant differences between visit survey scores for the two surgeons in the checklist (P = 0.91) and traditional groups (P = 0.18).

**Table 1:** Visit survey scores

Question (Did the discussion with your doctor…)	Domain	Traditional group mean score (95% CI)	Checklist group mean score (95% CI)	Mean difference (95% CI)
1. Help you understand the different treatment options	Knowledge	4.85 (4.73– 4.97)	4.63 (4.37– 4.89)	0.22 (-0.05 to 0.50)
2. Help you understand why surgery is necessary	Knowledge	4.85 (4.73– 4.97)	4.85 (4.68– 5.02)	0.99 (-0.21 to 0.21)
3. Help you understand what will be done during surgery	Knowledge	4.88 (4.77– 4.99)	4.81 (4.66– 4.96)	0.48 (-0.12 to 0.25)
4. Help you understand the risks of surgery	Knowledge	4.82 (4.67– 4.97)	4.81 (4.66– 4.96)	0.94 (-0.21 to 0.23)
5. Help you understand the care required after surgery	Knowledge	4.82 (4.67– 4.97)	4.67 (4.46– 4.88)	0.24 (-0.10 to 0.42)
6. Result in you feeling satisfied with the visit	Satisfaction	4.85 (4.73– 4.97)	4.78 (4.58– 4.97)	0.52 (-0.15 to 0.30)
7. Answer all of your questions to your satisfaction	Satisfaction	4.91 (4.78– 5.04)	4.81 (4.66– 4.96)	0.34 (-0.10 to 0.30)
8. Seem relaxed and free of pressure	Experience	4.88 (4.77– 4.99)	4.54 (4.36– 4.72)	0.05 (0.01 to 0.42)
9. Feel like your doctor wanted you to understand all aspects of the surgery	Experience	4.85 (4.70– 5.00)	4.81 (4.66– 4.96)	0.72 (-0.18 to 0.25)
10. Help you feel more certain about the decision for your child to have surgery	Decisional conflict	4.79 (4.63– 4.95)	4.74 (4.54– 4.94)	0.68 (-0.20 to 0.31)
11. Increase the level of trust you have in your surgeon	Decisional conflict	4.88 (4.74– 5.02)	4.78 (4.59– 4.97)	0.39 (-0.13 to 0.34)

## Discussion

The deficiencies of the informed consent process are striking [4,9,15]. For example, Weeks et al. found that only 19% of patients receiving chemotherapy for metastatic colorectal cancer knew that the treatment was not curative [4]. In the pediatric setting, Wasserzug et al. found that only 48% of parents responded correctly to at least one of two essential postoperative care questions (how to address bleeding from the mouth and what to feed their child) one hour after informed consent for tonsillectomy was completed [8]. One simple explanation for the inability to achieve adequate comprehension is that key items and steps are often left out of the consent process [16,17]. McGaughey evaluated 50 patients undergoing knee arthroscopy and identified that information regarding recovery was provided to only 30% of patients [17]. Braddock et al. found that patient understanding during consent discussions for orthopaedic procedures was verified only 12% of the time [16]. 

These findings suggest that the informed consent process is an ideal application for checklist use. However, our hypothesis that asking families to complete a checklist to verify their understanding of the surgeon would result in improvements in knowledge, satisfaction, patient experience, and decisional conflict during the informed consent process was not supported. While the checklist can identify different aspects of surgery that require further explanation, knowledge acquisition may primarily be influenced by supporting materials such as videos or the quality of the explanation [8,18-20]. It is also likely that satisfaction and experience are more influenced by effective communication, minimal waiting times, and a pleasant office environment than use of a checklist [21]. Finally, the checklist may not have impacted decisional conflict as this is more likely resolved before the surgery and preoperative visit are scheduled. 

The identification of two families (7.4%) on the checklist assessment questionnaire who strongly disliked the checklist and the traditional group reporting that the visits were significantly more relaxed suggests that the checklist was negatively perceived by some families. The negative perception may be secondary to form fatigue from the multiple documents that require completion at a preoperative appointment. Families may also feel that answering these questions before seeing the provider is unnecessary as they can simply be asked during the visit.

Limitations of this study include a sample size that was limited to patients seen for preoperative visits at the main campus location only. In addition, while we were able to identify that there were no significant differences in visit scores between surgeons, we did not evaluate the impact of other potential confounders such as the use of pictures to describe the surgery, the type of procedure being discussed, patient age, and the time from the last office visit to the preoperative appointment. It also may have been helpful to collect socioeconomic and educational background information from the families. The results are also subject to bias from the quality of the physician’s preoperative discussions, as the presence or absence of the consent checklist made it impossible to blind the physicians to the study group for the entire visit. Lastly, it would have been helpful to evaluate task completion, which is a main feature of the checklist. Comparing how often the critical components of informed consent (options, indications, technique, recovery, risks, benefits) were discussed in each group would likely have demonstrated an advantage in the checklist group based on the known deficiencies using traditional processes [16,17]. 

As the checklist completed by families did not improve the evaluated domains of the consent process and was perceived poorly by some families, it may be better to have the physician use the checklist without providing it to families. A physician-completed checklist would ensure that all aspects of surgery are equally covered, minimizing surgeon bias to cover what they are most interested in (how the surgery is done) and neglect what the patient is most interested in or has the most questions about (recovery) [22]. This method would also minimize form fatigue in families while providing standardization to the process. 

**Figure 5:**
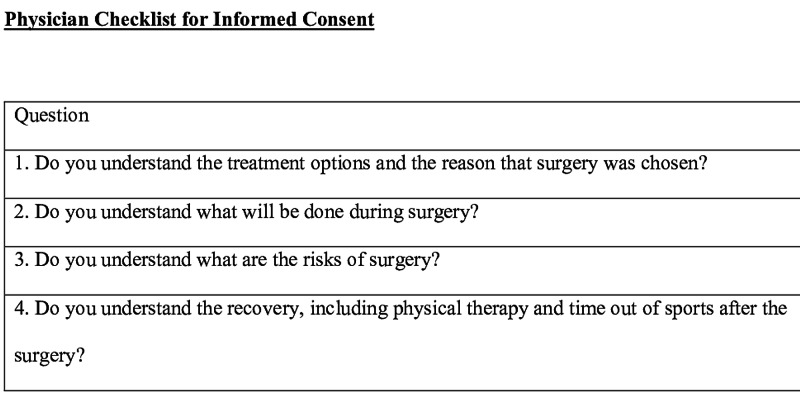
Proposed checklist to be completed by the physician during the informed consent process

We have developed a physician-completed checklist for informed consent that is applicable to any type of procedure, verifies appropriate aspects of the consent process, and can be placed in the medical record (Figure 5). Additional items such as assessment of level of comfort with the proceeding, the role of residents, and cost are worthy of consideration for inclusion as well [23]. This checklist should serve as an adjunct to materials such as decision aids and consent videos [24].

## Conclusions

Our results reflect the impact of a checklist on a defined informed consent process and may not be generalizable for other settings. Another common way of conducting a preoperative visit is to have the child see a physician extender for a history and physical, and then to see the surgeon again on the day of surgery to sign the consent form. However, this practice may decrease as hospitals begin to require surgery consents be completed prior to the day of surgery. While it may be assumed that this or other processes are adequate, we believe that the importance of informed consent and its known shortcomings suggest that we measure and continuously improve this process to ensure its purpose is being served. Further research is indicated to determine if the proposed physician checklist assists in completing the tasks of information disclosure and improves other domains of the consent process. The informed consent process will also benefit from further research into methods to improve patient recall and to develop simple assessment tools to assist with continuous improvement.
